# Venetoclax causes metabolic reprogramming independent of BCL-2 inhibition

**DOI:** 10.1038/s41419-020-02867-2

**Published:** 2020-08-13

**Authors:** Alba Roca-Portoles, Giovanny Rodriguez-Blanco, David Sumpton, Catherine Cloix, Margaret Mullin, Gillian M. Mackay, Katelyn O’Neill, Leandro Lemgruber, Xu Luo, Stephen W. G. Tait

**Affiliations:** 1 Cancer Research UK Beatson Institute, University of Glasgow, Garscube Estate, Switchback Road, Glasgow, G61 1QH UK; 2 Institute of Cancer Sciences, University of Glasgow, Garscube Estate, Switchback Road, Glasgow, G61 1QH UK; 3 Glasgow Imaging Facility, Institute of Infection, Immunity and Inflammation, University of Glasgow, University Avenue, Glasgow, G12 8QQ UK; 4 Eppley Institute for Research in Cancer and Allied Diseases, Fred and Pamela Buffett Cancer Center, University of Nebraska Medical Center, Omaha, NE 68198 USA

**Keywords:** Metabolomics, Apoptosis

## Abstract

BH3-mimetics are a new class of anti-cancer drugs that inhibit anti-apoptotic Bcl-2 proteins. In doing so, BH3-mimetics sensitise to cell death. Venetoclax is a potent, BCL-2 selective BH3-mimetic that is clinically approved for use in chronic lymphocytic leukaemia. Venetoclax has also been shown to inhibit mitochondrial metabolism, this is consistent with a proposed role for BCL-2 in metabolic regulation. We used venetoclax to understand BCL-2 metabolic function. Similar to others, we found that venetoclax inhibited mitochondrial respiration. In addition, we also found that venetoclax impairs TCA cycle activity leading to activation of reductive carboxylation. Importantly, the metabolic effects of venetoclax were independent of cell death because they were also observed in apoptosis-resistant BAX/BAK-deficient cells. However, unlike venetoclax treatment, inhibiting BCL-2 expression had no effect on mitochondrial respiration. Unexpectedly, we found that venetoclax also inhibited mitochondrial respiration and the TCA cycle in BCL-2 deficient cells and in cells lacking all anti-apoptotic BCL-2 family members. Investigating the basis of this off-target effect, we found that venetoclax-induced metabolic reprogramming was dependent upon the integrated stress response and ATF4 transcription factor. These data demonstrate that venetoclax affects cellular metabolism independent of BCL-2 inhibition. This off-target metabolic effect has potential to modulate venetoclax cytotoxicity.

## Introduction

Anti-cancer therapies often kill cancer cells through the mitochondrial (intrinsic) pathway of apoptosis^1^. In this process, mitochondrial outer membrane permeabilisation (MOMP) is the key initiating event for cell death^2^. MOMP allows the release of mitochondrial proteins, including cytochrome *c*, that activate caspase proteases and apoptosis. Because of its central role in regulating life and death, MOMP is tightly regulated, primarily by BCL-2 protein family members. The BCL-2 family is comprised of pro-apoptotic BAX, BAK and BH3-only proteins and anti-apoptotic BCL-2 members^3^. Anti-apoptotic BCL-2 proteins prevent MOMP and cell death by binding activated BAX, BAK and BH3-only proteins^4^.

Cancer cells often require increased anti-apoptotic BCL-2 expression to counteract pro-apoptotic stress, a state of increased apoptotic priming^5^. Because of the central role of BCL-2 proteins in preserving cancer cell viability, intense effort has centred upon developing drugs that neutralize anti-apoptotic Bcl-2 function. This has led to a new class of drugs called BH3-mimetics^6^. As their name implies, BH3-mimetics act similarly to BH3-only proteins, binding anti-apoptotic BCL-2 proteins in their hydrophobic groove thereby blocking their anti-apoptotic function. Various BH3-mimetics have been developed that target select or multiple members of the BCL-2 protein family. To date, venetoclax (also called ABT-199) represents the archetypal BH3-mimetic^7^. Developed as a specific inhibitor of BCL-2, venetoclax is now clinically approved to treat chronic lymphocyte leukaemia (CML) and acute myeloid leukaemia (AML)^8,9^.

BCL-2 family proteins have been implicated in a variety of non-apoptotic processes, most notably metabolism. Regarding BCL-2, reports have shown that BCL-2 overexpression increases COX activity and therefore promotes mitochondrial respiration^10^. Conversely, suppression of BCL-2 (by shRNA) decreased oxidative phosphorylation (OXPHOS) activity in primary AML cells^11^. Venetoclax has also been shown to affect cellular metabolism, further implicating BCL-2 in the regulation of metabolism^11–13^. Importantly, these metabolic effects of venetoclax have recently been shown to modulate its efficacy^12–14^. Nevertheless, how BCL-2 regulates metabolism is unclear. Given this, we set out to define metabolic functions of BCL-2 using venetoclax as a tool compound. We found that venetoclax inhibited mitochondrial respiration and tricarboxylic acid (TCA) function independent of cell death. While no effect of BCL-2 loss on metabolism was observed, surprisingly, venetoclax was found to inhibit mitochondrial function completely independent of its target BCL-2. However, the metabolic effect of venetoclax was dependent on ATF4 transcription factor function. These results demonstrate that venetoclax can affect cellular metabolism independent of BCL-2 protein inhibition.

## Results

### Venetoclax affects metabolism independent of BAX/BAK-mediated cell death

**Fig. 1:**
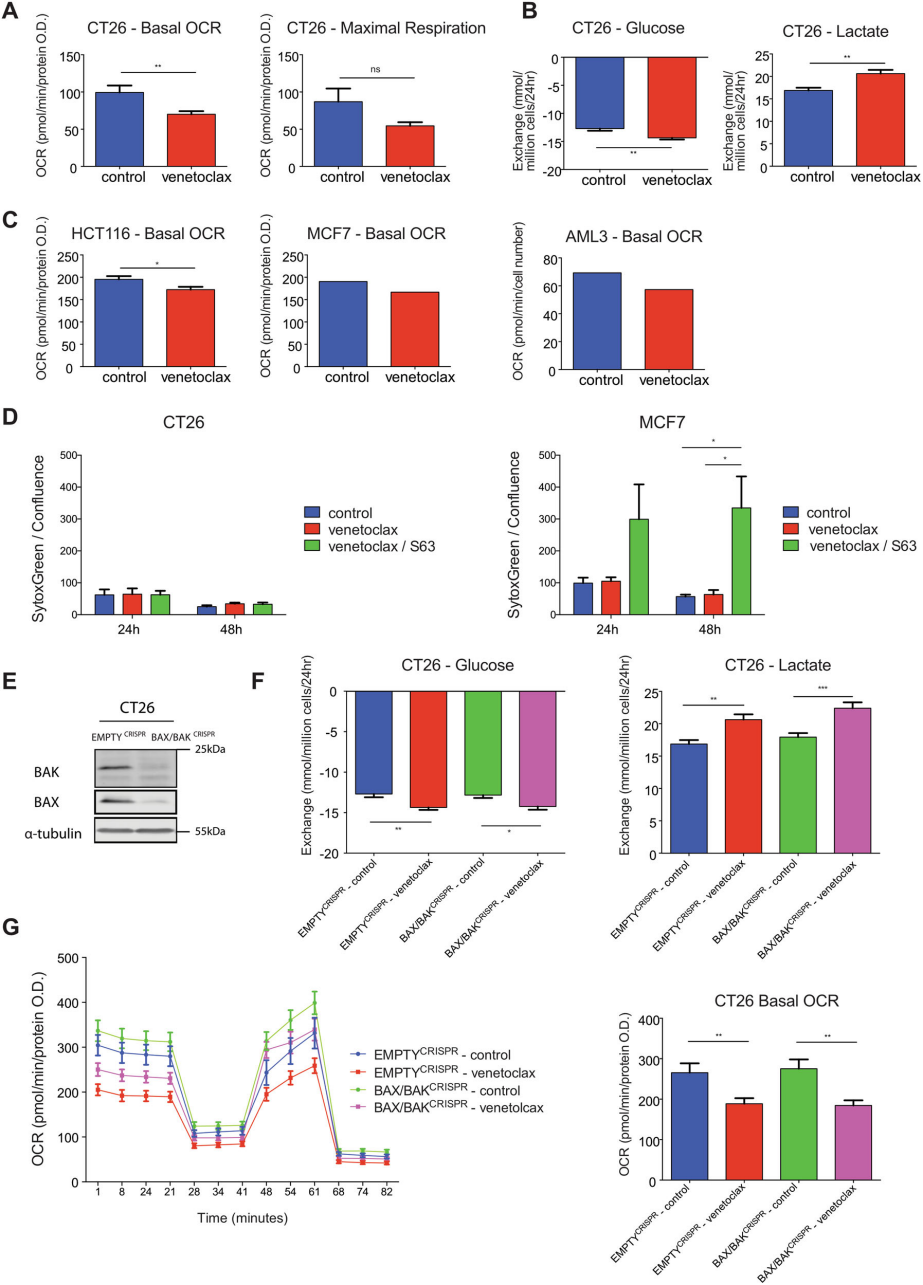
Venetoclax affects metabolism independent of BAX/BAK-mediated cell death. CT26 cells were treated with venetoclax (1 μM) for 24 h then oxygen consumption rate (OCR) was measured by Seahorse XF-96 Analyzer. **a** Basal OCR and maximal respiration. Data are the mean of three independent experiments ± SEM. **b** CT26 vector control cells were treated with venetoclax (1 μM) for 24 h, then glucose and lactate levels in the medium was measured by Biochemistry Analyzer YSI. Data represent the mean of four independent experiments ± SEM. **c** Basal OCR of HCT116 (mean of four independent experiments ± SEM), MCF7 (mean of one representative experiment, with six technical replicates, of two independent experiments), OCI-AML3 (one representative experiment, [mean of eight technical replicates], of two independent experiments). **d** CT26 or MCF7 cells were treated with venetoclax (1 μM) and S63845 (1 μM), alone or in combination for the indicated time points and cell viability was monitored with Sytox Green exclusion and Incucyte live-cell imaging. Data represent the mean of three independent experiments ± SEM. **e** Expression of BAX and BAK from CT26 CRISPR-EMPTY or CRISPR-BAX/BAK cells was measured by western blot, α-tubulin was included as a loading control. CT26 CRISPR-EMPTY or CRISPR-BAX/BAK were treated with venetoclax (1 μM) for 24 h, then metabolites were measured from media (**f**) and OCR was determined (**g**). Data represent the mean of four (**f**) or three (**g**) independent experiments ± SEM. Samples were compared using two-tailed, unpaired Student’s *t* test. *p < 0.05, **p < 0.01, ***p < 0.001.

Various studies implicate Bcl-2 family proteins in metabolic regulation. To understand how BCL-2 might regulate metabolism, we used venetoclax (also called ABT-199), a BCL-2 specific BH3-mimetic^7^. CT26 cells (murine colorectal cancer cell line) were treated with venetoclax (1 μM) for 24 h and analysed for mitochondrial respiratory function by Seahorse extracellular flux assay (Fig. 1a and Fig. S1A). Venetoclax treatment led to a decrease in CT26 basal oxygen consumption rate (OCR), indicating an inhibitory effect on mitochondrial OXPHOS (Fig. 1a). Maximal OCR (after treatment with the proton ionophore CCCP) was unaffected following venetoclax treatment (Fig. 1a). Cells can compensate for lower mitochondrial respiration by increasing glycolysis^15^. Consistent with upregulation of glycolysis, we observed an increased uptake of glucose and lactate secretion in medium following treatment of CT26 cells with venetoclax (24 h, 1 μM) (Fig. 1b). To define if the effects of venetoclax on OCR were immediate, CT26 cells were treated with venetoclax (1 μM) and mitochondrial OCR was assessed over a 4-h time period. Venetoclax did not induce an immediate drop in OCR, however an effect became apparent after 4 h treatment (Fig. S1B). We extended our analysis to a panel of cell lines (human colorectal cancer; HCT116, human breast cancer; MCF7 and human AML; OCI-AML-3) analysing mitochondrial respiratory function by Seahorse assay following venetoclax treatment (24 h, 1 μM) (Fig. 1c). In all cases, basal OCR was reduced following venetoclax treatment, indicating that venetoclax inhibition of mitochondrial function is independent of cell types tested. During apoptosis, MOMP is associated with a rapid loss of mitochondrial respiratory function^16^. Consequently, the metabolic effects of venetoclax treatment might be secondary to cell death caused by neutralisation of anti-apoptotic BCL-2 function. To investigate this possibility, CT26 and MCF7 cells were treated with venetoclax (1 μM) and analysed for cell viability by Sytox Green exclusion and Incucyte live-cell analysis (Fig. 1d). Treatment with venetoclax alone had no impact on cell viability over a 48-h period in both cell lines whereas combined treatment of venetoclax with S63845 (MCL-1 targeting BH3-mimetic)^17^ induced cell death in MCF7 cells (Fig. 1d). This result argues that the metabolic effects of venetoclax are independent of cell death. To definitively address this, we used CRISPR/Cas9 genome editing to generate CT26 cells deficient in BAX and BAK—two proteins that are essential for mitochondrial apoptosis (Fig. 1e)^18,19^. As expected, BAX/BAK deleted CT26 cells were completely resistant to a combination of BH3-mimetics (ABT-737 and S63845, 10 and 1 μM, respectively, added to inhibit BCL-2, BCL-xL, BCL-W and MCL-1) (Fig. S1C). Using these cells, we analysed the effect of venetoclax treatment in metabolism by analysis of extracellular metabolite levels (Fig. 1f) and mitochondrial respiratory function (Fig. 1g). Venetoclax treatment led to a decrease in basal OCR, and increase in glucose uptake and lactate secretion independent of BAX and BAK. These data demonstrate that the BCL-2 targeted BH3-mimetic venetoclax can affect cellular metabolism independent of its canonical pro-apoptotic function.

### Venetoclax inhibits the TCA cycle causing reductive carboxylation

**Fig. 2:**
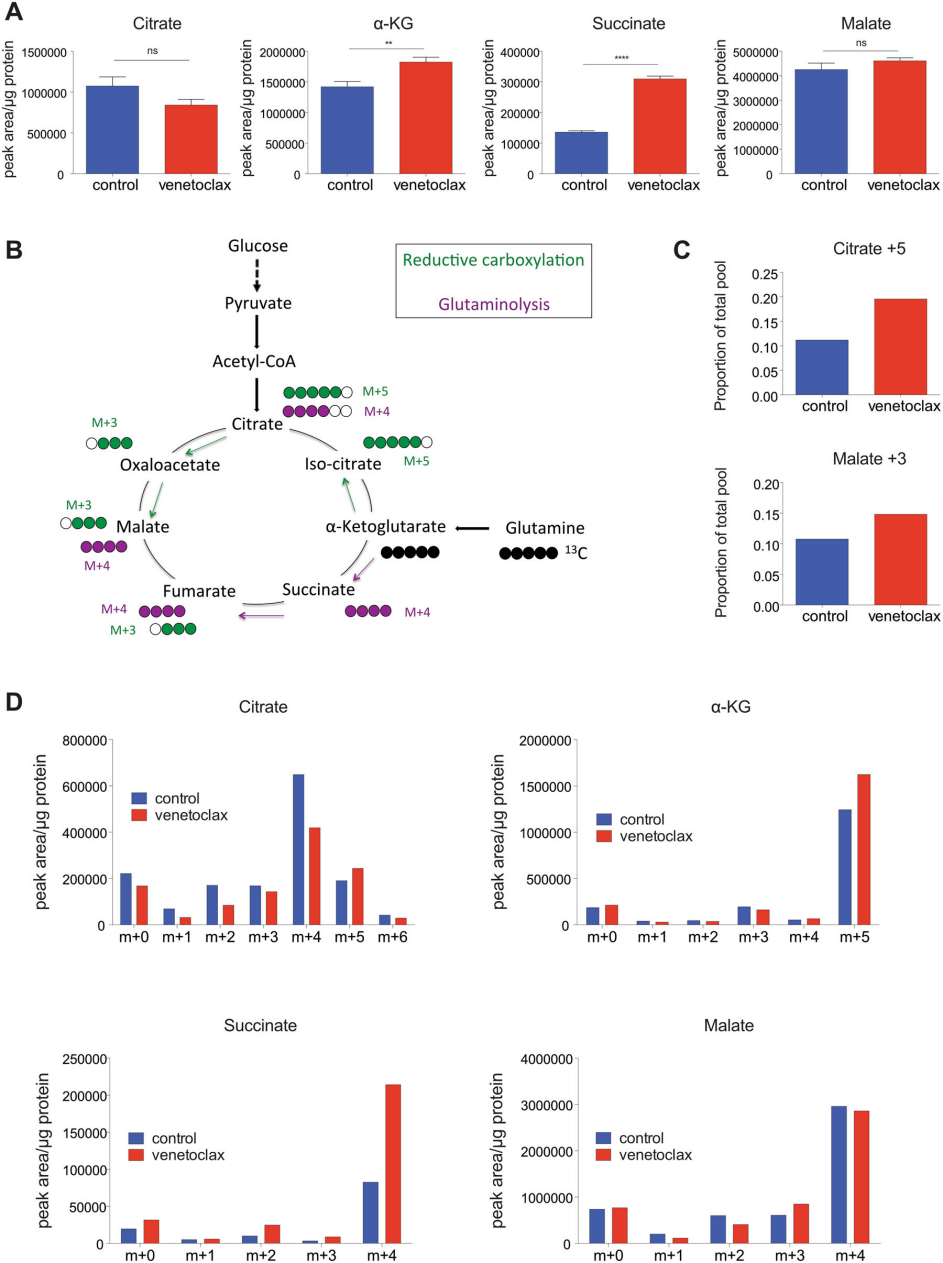
Venetoclax inhibits the TCA cycle causing reductive carboxylation. **a** CT26 cells were treated for 24 h with venetoclax (1 μM) and intracellular metabolites were extracted and analysed by LC–MS. Total levels of metabolites are shown from three independent experiments ± SEM. **b** Diagram showing carbon labelling when cells are cultured with ^13^C-glutamine. **c**, **d** CT26 cells were treated for 24 h with venetoclax (1 μM) and cultured with labelled glutamine (^13^C-GLN). Labelled metabolites from three technical replicates of one representative experiment (out of two). Samples were compared using two-tailed, unpaired Student’s *t* test. *p < 0.05, **p < 0.01, ***p < 0.001.

We aimed to understand how venetoclax affects metabolism. Because we had detected alterations in glucose uptake and lower mitochondrial respiration following venetoclax treatment, we focused on metabolites involved in the TCA cycle, also known as the citric acid cycle. The TCA cycle is a series of redox reactions carried out in the mitochondrial matrix mainly supported by two carbon sources: (1) glucose, which provides carbons, through its glycolytic conversion into pyruvate and then into acetyl-CoA and (2) glutamine that is converted into glutamate and then into α-ketoglutarate (α-KG). To investigate potential effects of venetoclax on the TCA cycle, CT26 cells were treated with venetoclax (1 μM, 24 h) and intracellular metabolite levels were analysed by LC–MS (Fig. 2a). A variety of TCA metabolite levels were altered following venetoclax treatment, specifically α-KG and succinate levels were increased while citrate and malate were unaltered (Fig. 2a). Glutamine enters the citric acid cycle as α-KG (via glutaminolysis) and is then converted to succinate. Alternatively, through reductive carboxylation, the TCA cycle can operate in reverse, converting α-KG to citrate (Fig. 2b). The succinate accumulation that we observed suggested an inhibition in TCA cycle, therefore, to analyse TCA function, we employed stable isotope tracing using labelled ^13^C-glucose or ^13^C-glutamine. The respective labelled metabolites were added with or without venetoclax treatment (1 μM, 24 h) to CT26 cells and intracellular metabolites were analysed by LC–MS. Higher label incorporation was found in TCA cycle metabolites using ^13^C-glutamine (Fig. S2A) compared with ^13^C-glucose (Fig. S2B), indicating that glutamine is the main substrate for the TCA cycle in these cells. Consequently, we analysed different metabolites following ^13^C-glutamine labelling. This analysis showed that venetoclax treatment increased the levels of glutamate m+5 (Fig. S2C) and α-KG m+5 (Fig. 2d and Fig. S2A) demonstrating increased glutamine metabolism. In line with this, the increase of total succinate levels was found mainly as succinate m+4 (Fig. 2d and Fig. S2A). Consistent with an inhibition of the TCA cycle by venetoclax, we also observed an increased in labelled citrate m+5 and malate m+3, indicative of reductive carboxylation (Fig. 2c, d and Fig. S2A). Collectively these data demonstrate that venetoclax inhibits the mitochondrial TCA cycle, and suggest an activation of reductive carboxylation upon treatment.

### Metabolic effects of venetoclax are independent of Bcl-2 protein inhibition

**Fig. 3:**
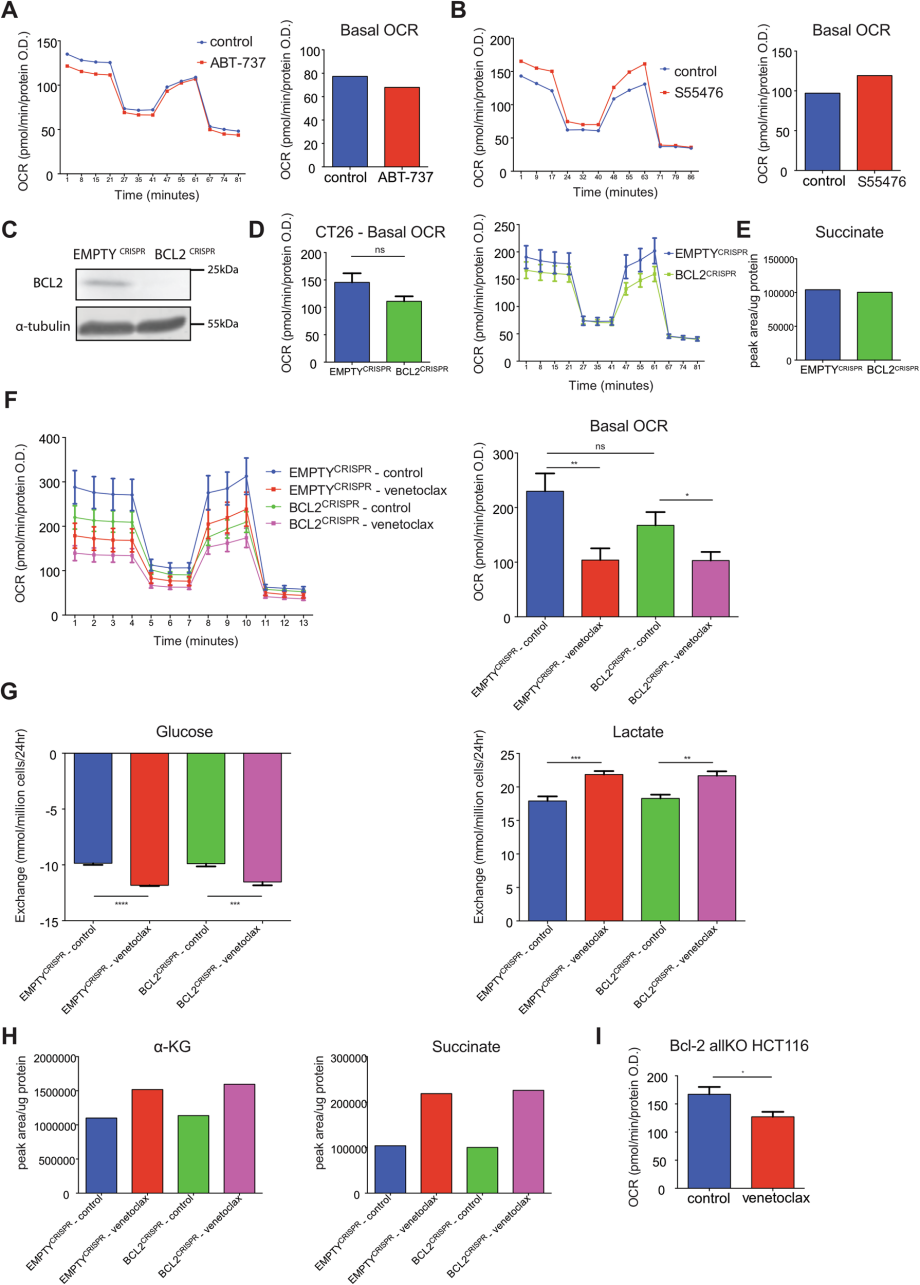
The metabolic effects of venetoclax are independent of BCL-2 family proteins. **a** CT26 cells were treated for 24 h with ABT-737 (1 μM) and OCR was measured. Data represent the mean of five technical replicates from one representative experiment (out of two). **b** CT26 cells were treated for 24 h with S55746 (1 μM) and OCR was measured. Data represent the mean of five technical replicates from one representative experiment (out of two). **c** BCL-2 protein expression in CT26 CRISPR-EMPTY and CT26 CRISPR-BCL2 cells was determined by western blot, α-tubulin was probed as a loading control. **d** OCR of CT26 CRISPR-EMPTY and CT26 CRISPR-BCL2 cells. Data represent the mean of five independent experiments ± SEM. **e** Metabolite levels of CT26 CRISPR-EMPTY and CT26 CRISPR-BCL2 cells. Data represent the mean of three technical replicates. CT26 CRISPR-EMPTY and CT26 CRISPR-BCL2 cells were treated with venetoclax (1 μM) for 24 h; OCR was assessed (**f**), metabolites from the media were measured (**g**), and intracellular metabolites were extracted and analysed by LC–MS (**h**). Data represent the mean of three independent experiments ± SEM (**f**, **g**) or one independent experiment with three technical replicates (**h**). **i** Basal OCR of Bcl-2 allKO HCT116 cells after treatment with venetoclax (1 μM) for 24 h. Data represent the mean of four independent experiments ± SEM. Samples were compared using two-tailed, unpaired Student’s *t* test. *p < 0.05, **p < 0.01, ***p < 0.001 and ****p < 0.0001.

Because venetoclax is a BCL-2 specific BH3-mimetic, our data implicated BCL-2 in the regulation of metabolism. To investigate this, we determined whether targeting BCL-2 with alternative BH3-mimetics, S55746 (specific for BCL-2)^20^ or ABT-737 (targeting BCL-2, BCL-xL and BCL-W)^21^ had similar metabolic effects. CT26 cells were treated with ABT-737 or S55746 (1 μM for 24 h) and analysed for OCR by Seahorse assay (Fig. 3a, b). Unlike venetoclax, neither ABT-737 nor S55746 decreased OCR. In a second approach, we performed metabolic tracing using labelled ^13^C-glutamine, with or without S55746 treatment. In contrast to venetoclax (Fig. 2), S55746 treatment did not affect the labelling or levels of TCA metabolites (Fig. S3A). Surprisingly, these data demonstrate that additional BH3-mimetics that target BCL-2 do not phenocopy the metabolic effects of venetoclax. We reasoned this disparity in two ways; (1) either venetoclax binds BCL-2 in a distinct manner that affects metabolism or (2) venetoclax affects metabolism independent of BCL-2 binding. To discern these possibilities, we used CRISPR/Cas9 genome editing to generate BCL-2 deleted CT26 cells (Fig. 3c) and BCL-2 deleted SVEC4-10 (SV40 immortalised murine endothelial cell line) (Fig. S3B). Deletion of BCL-2 itself had no effect on basal OCR, determined by Seahorse assay (Fig. 3d and Fig. S3B), or TCA metabolite levels (Fig. 3e and Fig. S3B). We next treated BCL-2 deleted CT26 cells with venetoclax and assessed OCR (Fig. 3f). Crucially, venetoclax also inhibited OXPHOS in BCL-2 deleted cells. Extending this, we analysed extracellular metabolite levels (Fig. 3g) or TCA metabolites (Fig. 3h) in BCL-2 deleted cells following venetoclax treatment. An increase in glucose uptake and lactate release was observed following treatment, irrespective of BCL-2 expression (Fig. 3g). Moreover, similar to what we had observed previously, venetoclax induced an increase in α-KG and succinate levels that, again, was independent of BCL-2 (Fig. 3h). As an alternative approach, RNAi was used to inhibit BCL-2 expression in CT26 cells (Fig. S3C). Following venetoclax treatment, succinate levels were again increased and mitochondrial respiration was also decreased, irrespective of BCL-2 expression (Fig. S3D, E). To expand these findings, we generated BCL-2 deleted B16F10 murine melanoma cells using CRISPR/Cas9 genome editing (Fig. S3F). These cells were treated with venetoclax (1 μM, 24 h) and OCR was analysed by Seahorse assay (Fig. S3G) or TCA metabolite levels by LC–MS (Fig. S3H). Irrespective of BCL-2 expression, venetoclax inhibited mitochondrial OCR and increased α-KG and succinate levels. These data demonstrate that venetoclax affects cellular metabolism independently of its pro-survival target BCL-2. Finally, as venetoclax still has some affinity, albeit significantly lower, for other Bcl-2 proteins^7^ we investigated whether venetoclax effects on mitochondrial respiration were dependent on other anti-apoptotic BCL-2 family members. For this purpose, we used HCT116 cells deficient in all Bcl-2 family members, except BOK - called Bcl-2 allKO HCT116^22^. Bcl-2 allKO HCT116 cells were treated with venetoclax and analysed for OCR by Seahorse assay (Fig. 3i). Venetoclax also inhibited basal OCR in this setting. These data demonstrate that venetoclax affects cellular metabolism independent of its cognate target BCL-2 and Bcl-2 family proteins in general.

### Venetoclax affects mitochondrial morphology and function

**Fig. 4:**
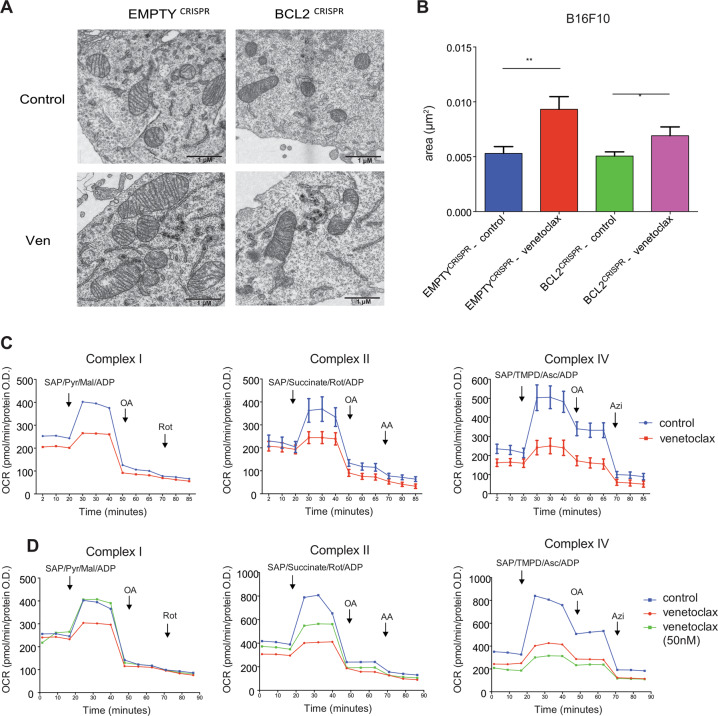
Venetoclax affects mitochondrial morphology and function. **a** TEM images from B16F10 CRISPR-EMPTY and CRISPR-BCL2 cells after 24 h of venetoclax (1 μM) treatment. Scale bar = 1 μm. **b** A random selection of at least 25 mitochondria across different cells and fields was analysed for each condition from images in **a**. Mitochondrial area (in μm^2^) was quantified using ImageJ software. Data represent the mean of at least 25 mitochondria ± SEM. **c** CT26 cells were treated for 24 h with venetoclax (1 μM), then activity of complexes I, II and IV was assessed by Seahorse XF-96 Analyzer. Complex I data represent the mean of two independent experiments; complex II data represent the mean of four independent experiments ± SEM and complex IV data represent the mean of three independent experiments ± SEM. **d** CT26 cells were treated for 24 h with vehicle, venetoclax (1 μM) or venetoclax (50 nM), then activity of complexes I, II and IV was assessed. Data represent the mean of one independent experiment with four technical replicates. Samples were compared using two-tailed, unpaired Student’s *t* test. *p < 0.05, **p < 0.01, ***p < 0.001. Ven = venetoclax.

We next sought to understand how venetoclax affected metabolism independent of BCL-2. Due to the effects previously observed (reduced OCR and inhibition of TCA cycle), we first asked whether venetoclax treatment affected mitochondrial morphology. Control or BCL-2 deleted B16F10 cells were treated with venetoclax (1 μM, 24 h) and mitochondrial morphology was analysed by electron microscopy (EM) (Fig. 4a, b). While BCL-2 deletion had no effect on mitochondrial morphology, venetoclax treatment led to mitochondrial enlargement independent of BCL-2 (Fig. 4a, b). To extend this finding, we also analysed mitochondrial morphology in another cell line, MCF7, following venetoclax treatment (1 μM, 24 h) (Fig. S4). Treatment with venetoclax (1 μM, 24 h) also led to aberrant mitochondrial morphology in MCF7 cells. Because venetoclax affected mitochondrial morphology, we investigated whether venetoclax could affect mitochondrial activity, focusing on electron transport chain function. CT26 cells were treated with venetoclax (1 μM, 24 h) and the activity of respiratory complexes I, II and IV was measured by Seahorse analysis (Fig. 4c). Importantly, venetoclax inhibited the activity of all respiratory complexes tested (Fig. 4c). Finally, we investigated effects of venetoclax dose dependency. CT26 cells were treated for 24 h with varying concentrations of venetoclax (50 nM–1 μM) and assayed for complex activity using Seahorse assay (Fig. 4d). At 1 μM venetoclax, the function of all respiratory complexes was affected; however, at a lower dose (50 nM), complex I activity was unaffected, whereas complex IV activity was still inhibited, and complex II only partially (Fig. 4d). Collectively, these data demonstrate that venetoclax alters mitochondrial morphology independent of BCL-2 inhibition and broadly inhibits mitochondrial respiratory complex function.

### Venetoclax affects metabolism dependent on ATF4

**Fig. 5:**
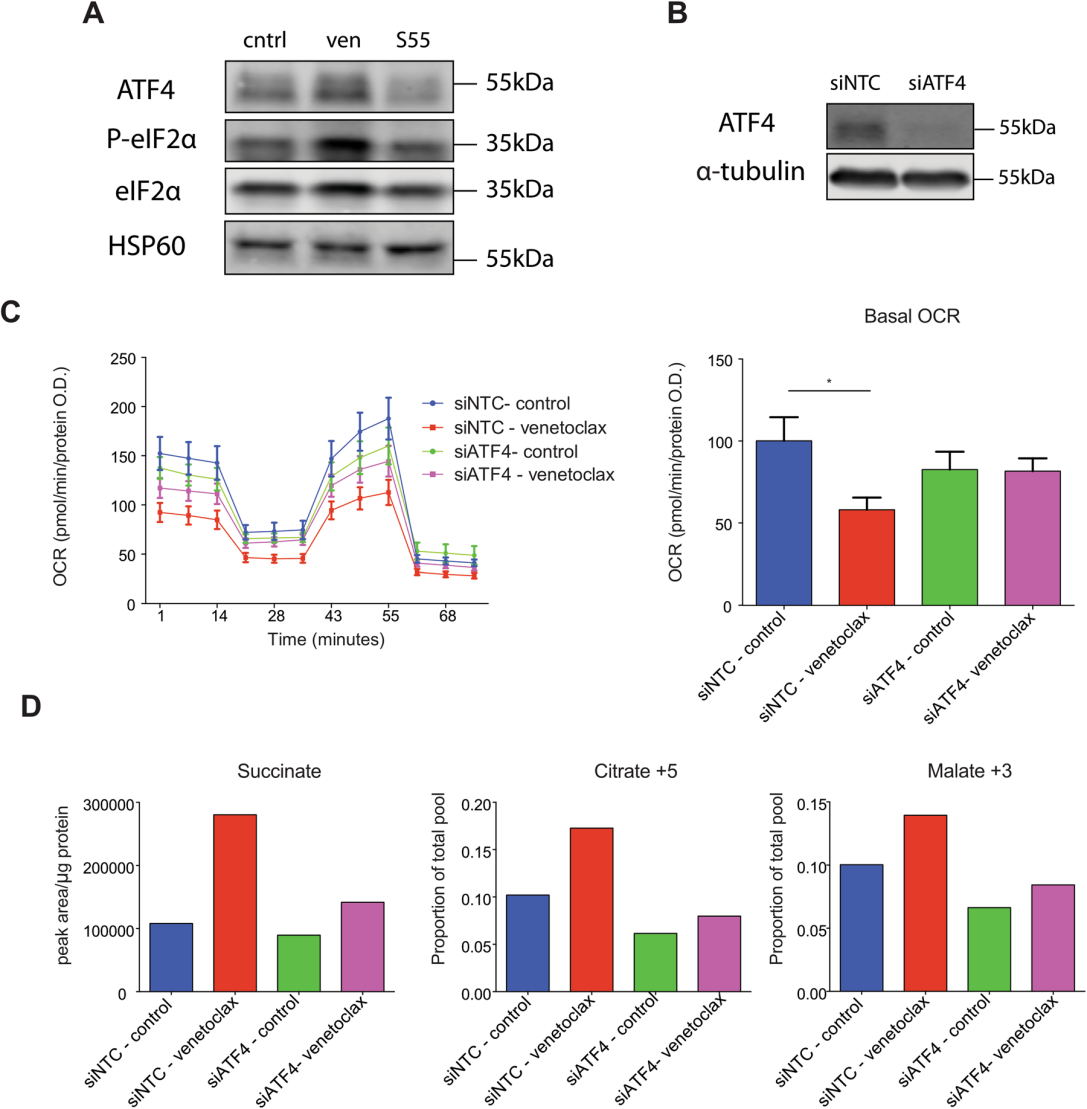
Venetoclax affects metabolism dependent on ATF4. **a** Expression of ATF4, P-eIF2α and eIF2α from CT26 cells treated with venetoclax or S55746 (1 μM, 24 h) was measured by western blot, HSP60 was included as a loading control on the same blot. **b** ATF4 protein expression in CT26 cells following transfection with non-targeting siRNA (NTC) or siRNA against *Atf4* was determined by western blot, α-tubulin was probed as a loading control on the same blot. **c** NTC or ATF4 siRNA transfected CT26 cells were treated with venetoclax (1 μM) for 24 h and assessed for OCR. Data represent the mean of three independent experiments ± SEM. **d** NTC or ATF4 siRNA transfected CT26 cells were treated with venetoclax (1 μM) for 24 h and cultured with labelled glutamine (^13^C-GLN). Intracellular metabolites were extracted and analysed by LC–MS. Graphs represent total succinate levels and relative citrate m+5 and malate m+3 levels of one independent experiment with three technical replicates. Samples were compared using two-tailed, unpaired Student’s *t* test. *p < 0.05, **p < 0.01, ***p < 0.001. Ven = venetoclax, S55 = S55746.

We next investigated how venetoclax could affect mitochondrial ETC function. Given its broad effects on complexes I, II and IV activity, we reasoned that venetoclax may impact on respiratory complex assembly. To investigate this, MCF7 cells were treated with venetoclax (1 μM, 24 h) and ETC complex integrity was assessed by blue native gel (Fig. S5). However, venetoclax had no effect on respiratory complex assembly. We had previously observed that venetoclax effects on oxygen consumption are not immediate (Fig. S1B). This delayed effect might be due to venetoclax regulating OXPHOS in an indirect manner. Mitochondrial stress can elicit a process called the integrated stress response (ISR) characterised by phosphorylation of eIF2α and upregulation of the transcription factor ATF4^23^. Consequently, we investigated whether venetoclax treatment led to ISR activation. CT26 cells were treated with venetoclax or S55746 (1 μM, 24 h) and analysed by for phospho-eIF2α and ATF4 levels by western blot (Fig. 5a). Importantly, venetoclax treatment increased phospho-eIF2α and ATF4 levels, consistent with engagement of the ISR, whereas S55746 had no effect. ATF4 is known to regulate metabolic adaptation through the transcription of various metabolic enzymes^24^. We therefore investigated whether venetoclax altered mitochondrial metabolism in an ATF4-dependent manner. To this end, we used RNA interference to effectively supress ATF4 expression in CT26 cells (Fig. 5b). Using this approach, we assessed the requirement for ATF4 in venetoclax-induced mitochondrial OXPHOS inhibition (Fig. 5c). Importantly, ATF4 loss prevented the venetoclax-induced reduction in OCR. To study this further we investigated effects of venetoclax on TCA metabolites with or without ATF4 (Fig. 5d). In line with our previous findings, venetoclax induced increases in succinate was partially rescued by depletion of ATF4. In addition, activation of reductive carboxylation induced by venetoclax (assessed by increase in citrate m+5 and malate m+3) was also decreased upon loss of ATF4 expression. In sum, these data demonstrate that venetoclax activates the ISR, leading to ATF4-dependent metabolic reprogramming.

## Discussion

Venetoclax is a potent, clinically approved BH3-mimetic that induces apoptosis by targeting BCL-2^7^. Recent studies have shown that venetoclax also has metabolic effects, thereby implicating BCL-2 in the regulation of metabolism^11–14^. Our initial goal was to use venetoclax to understand the metabolic functions of BCL-2. Similar to others, we also found that venetoclax exerts metabolic effects—decreasing mitochondrial respiration and inhibiting the TCA cycle. The metabolic effects of venetoclax were found to be independent of the canonical, pro-survival function of BCL-2, because they were also observed in apoptosis-resistant BAX/BAK-deficient cells. Crucially, venetoclax also affected mitochondrial metabolism in BCL-2 deficient cells and, indeed, in cells lacking all anti-apoptotic Bcl-2 family members. This demonstrates that venetoclax can affect metabolism independent of its BCL-2 inhibitory function. Investigating the underlying basis for these metabolic effects, we found that they were dependent on activation of the ISR and the transcription factor ATF4.

Using apoptosis-resistant BAX/BAK-deficient cells, our data definitively show that venetoclax can decrease mitochondrial respiration independent of cell death. Supporting this view, other studies have found that venetoclax can inhibit respiratory function in the absence of obvious cell death^12,13^. Nevertheless, a recent study has shown that venetoclax-impairment of OXPHOS at short-time points post-treatment requires BAX/BAK-dependent MOMP^14^. This is somewhat expected, given the well-established inhibitory effects of MOMP on mitochondrial respiration^16^. Importantly, the cell death (BAX/BAK independent) effects of venetoclax treatment we observed occurred upon prolonged treatment. This is consistent with our finding that the ISR and ATF4 transcriptional activity are required for venetoclax dependent metabolic effects.

Our study’s rationale was to use venetoclax to probe how BCL-2 could regulate metabolism. Various studies have implicated a role for BCL-2 in the regulation of mitochondrial metabolism^10,11^. However, during our investigation of venetoclax activity, we failed to observe any effect of suppressing BCL-2 expression (either via RNAi or CRISPR/Cas9 genome editing) on mitochondrial function in different cell lines. There could be many reasons for this, including artefactual effects of BCL-2 overexpression upon mitochondrial function, cell type specific effects and/or indirect effects of BCL-2 affecting metabolism, for instance, through effects on cell viability.

Our surprising finding was that venetoclax could inhibit mitochondrial respiration independent of its target BCL-2. This is supported by different lines of evidence including data that other BH3-mimetics that target BCL-2, including S55746 and ABT-737, failed to exert any metabolic effect. Most conclusively, venetoclax also inhibited mitochondrial respiration in cells deficient in BCL-2 and, indeed, in cells devoid of all anti-apoptotic Bcl-2 proteins. Importantly, this precludes using venetoclax as tool to define BCL-2 metabolic effects.

A remaining question is how does venetoclax affect mitochondrial function independent of BCL-2? Towards answering this, we found that venetoclax affected mitochondrial morphology, again independent of its target BCL-2. Alterations in mitochondria morphology have been observed in several mitochondrial pathologies^25,26^. For instance, cancers that harbour mutations in the TCA cycle protein fumarate hydratase, present a disrupted TCA cycle^27^, impaired OXPHOS, increased glycolysis and abnormal mitochondrial morphology^28,29^, phenocopying the observed effects induced by venetoclax. Based on these and many other studies, we consider it likely that venetoclax-induced mitochondrial dysfunction leads to aberrant mitochondrial morphology. In addition, it has been reported that the shape of mitochondrial cristae determines the efficiency of mitochondrial respiration^30^, demonstrating a link between mitochondrial morphology and function. In line with this, analysis of electron transport chain function (complexes I, II, IV) showed that venetoclax displayed broad inhibitory activity. Corroborating our findings, others have recently shown that venetoclax can inhibit complex I activity^13^. Importantly, other studies have described synergy between ETC inhibitors with venetoclax treatment^14,31^. Thus, the respiratory effects of venetoclax we describe here may contribute to this synergistic effect. Nevertheless, we suspect that these effects on respiratory complex function are most likely indirect, due to the lag time between addition of venetoclax and inhibition of mitochondrial metabolism.

This prompted us to investigate alternative possibilities, where we found that the metabolic effects of venetoclax were dependent on the transcription factor ATF4. ATF4 has been described as the master regulator of mitochondrial stress, activated as part of the ISR^32^. Various studies have found that the ISR and ATF4 can be activated upon chemotherapy and contribute to chemoresistance^33–35^. Moreover, within the tumour microenvironment, stromal fibroblasts can also upregulate ATF4 to induce metabolic changes that promote tumour proliferation^36^. Nevertheless, ATF4 can have opposing activities; upon a mild stress, it can restore cellular homoeostasis, whereas upon an excessive or prolonged stress, ATF4 activity can cause cell death^23^. Along these lines, ATF4 has recently been shown to promote or circumvent resistance to venetoclax^13,31,37^. As such, the off-target ATF4 effect we describe here may have potential to modulate the effectiveness of venetoclax cytotoxicity.

## Materials and methods

### Cell culture

All cell lines were cultured at 37 °C in a humified incubator with 5% CO_2_. SVEC4-10, B16F10, MCF7, HCT116 and HEK-293FT cells were maintained in Dulbecco’s modified Eagle’s medium (DMEM), high glucose medium (25 mM). CT26, OCI-AML3 cells were cultured in RPMI medium. All media were supplemented with 10% FBS, 2 mM glutamine, 1 mM sodium pyruvate and 50 μM β-mercaptoethanol. All the cell lines were determined negative for mycoplasma.

### Cell viability assays

Cell viability was monitored by Incucyte imaging system (Incucyte FLR, Incucyte ZOOM or Incucyte S3 Live-Cell Analysis System from Essen Bioscience), using the non-cell-permeable nuclear stain 30 nM SYTOX Green (SG, S7020, Invitrogen), according to the manufacturer’s protocols. The number of SYTOX Green-positive cells was normalized to cell confluence in each well. In every experiment, two technical replicates were performed and experiment was independently repeated three times.

### Lentiviral transduction

HEK-293FT (2 × 10^6^) cells were co-transfected with the indicated target plasmids and the packaging plasmids psPAX (Addgene, 12260) and pCMV-VSV-G (Addgene, 8454), using Lipofectamine 2000 (Invitrogen) according to the manufacturer’s instructions.

After 2 days, the viral supernatant produced from HEK-293FT cells was filtered (0.45 μm) and used to infect selected cells, in the presence of 1 μg/mL polybrene. After two rounds of infection, positive CRISPR/CAS9-deleted cells were selected by antibiotic (10 μg/mL of blasticidin and 1 μg/mL puromycin) for all cell lines, except CT26, which were selected with 10 μg/mL of puromycin. Plasmids: CRISPR-guide sequences: murine BCL2 (AGAAGTCATCCCCAGCCCGG), murine BAX (CAACTTCAACTGGGGCCGCG), murine BAK (GCGCTACGACACAGAGTTCC).

### siRNA transfection

siRNA transfection was performed in a six-well plate (1 × 10^5^ cells per well). Cells were cultured in antibiotic-free medium (800 μL) and transfected with 200 μL of Optimem medium (Thermo Scientific) containing 20 or 50 nM non-targeting control (si scramble, D-001206-13-05, Dharmacon), siRNA-targeting *Bcl2* (siGENOME SMARTpool (mouse), M-063933-01-0005, Dharmacon), siRNA-targeting *Atf4* (siGENOME SMARTpool (mouse), M-042737-01-0005, Dharmacon) using Lipofectamine 2000 (Invitrogen) according to the manufacturer’s protocol. After 5 h of incubation, cells were washed with PBS and fresh medium containing antibiotic (10000 units/mL penicillin) was added.

### Western blotting

Cells were lysed in RIPA buffer (10 mM Tris-HCl (pH 7.4), 150 mM NaCl, 1.2 mM EDTA, 1% Triton, 0.1%SDS) for 20 min. Afterwards, protein lysates were centrifuged at maximum speed (21130 rcf) for 10 min, at 4 °C. Protein quantification was performed using Pierce™ BCA Protein Assay (Thermo Fisher, 23250) according to the manufacturer’s protocol. Then, SDS-containing loading buffer (NuPAGE™ LDS Sample Buffer, NP0007) and DTT were added to the lysates (final concentration of 1x and 10 mM, respectively) for protein denaturation. Samples were boiled for 5 min at 100 °C before loading onto the gel. Proteins were separated by SDS-PAGE in Bio-Rad Western blot chambers (80–120 V) and then transferred onto nitrocellulose membrane (100 V for 1–1:30 h). Membranes were incubated with the primary antibody (5% milk or BSA in TBS-T) overnight at 4 °C. Then, they were incubated with the secondary antibody (5% milk in TBS-T) (Li-COR Biosciences) for 1 h at room temperature in the dark. Protein detection was achieved by Odyssey^®^ Imaging Systems CLx (Li-COR Biosciences). Primary antibodies: HSP60 (Cell Signaling, 4870, 1/1000), ATF4 (D4B8) (Cell Signaling, 11815, 1/500), Phospho-eIF2α (Ser51) (D9G8) (Cell Signaling, 3398, 1/500), eIF2α (D7D3) (Cell Signaling, 5324, 1/500), BCL-2 (10C4) (Santa Cruz, sc-23960, 1/500), α-tubulin (Sigma, T5168: 1/1000), BAK (D4E4) (Cell Signaling, 12105, 1/1000), BAX (Cell Signaling, 2772, 1/1000). Control panels were generated by assessing the expression of a housekeeping gene (HSP60 or α-tubulin) on the same blot.

### Mitochondrial respiration experiments

For mitochondrial respiration experiments, 10,000 cells/well were seeded in XFe96 plates (Agilent). Next day, cells were treated during 24 h with DMSO or drug treatment. After 24 h of treatment, the medium was aspirated, and replaced with the XF Mito stress medium (DMEM or RPMI supplemented with 1% FBS, 10 mM glucose, 2 mM glutamine, and 1 mM pyruvate, pH 7.4). Cells in XF Mito stress medium were incubated at 37 °C in the absence of CO_2_ for 30–45 min. OCR was measured using the XFe96 Extracellular Flux Analyzer (Agilent) according to the manufacturer’s instructions. Baseline OCR measurements were determined before administration of oligomycin (1 μM) (port A). Then, CCCP (1.5 μM) was added in port B, and a combination of rotenone (1 μM) and antimycin A (1 μM) in port C. After seahorse assay, OCR measurements were normalized to the amount of protein per well. Protein O.D. was measured using Pierce™ BCA Protein Assay (Thermo Fisher, 23250) according to the manufacturer’s protocol. For suspension cells (OCI-AML3), the plate was first pre-coated with 25 μL of Cell-Tak (Corning™ Cell-Tak Cell and Tissue Adhesive; Fisher Scientific, 10317081) solution at 0.02 mg/mL in 0.1 M NaHCO3, pH 6–8. Then, 80,000 cells/well were seeded in XFe96 plates (Agilent) in 50 μL of XF Mito stress medium (RPMI supplemented with 1% FBS, 10 mM glucose, 2 mM glutamine, and 1 mM pyruvate, pH 7.4). Then, the plate was centrifuged at 200 × *g* for 1 min, without brakes and incubated for 20–30 min at 37 °C in a CO_2_-free incubator. After incubation, 100 μL of seahorse medium was added to each well. Finally, the plate was incubated during another 20 min more at 37 °C in a CO_2_-free incubator. Seahorse analysis was carried out as described previously. Cell number was used for normalization of OCR values. Data analysis was conducted with the Seahorse Wave software version 2.2.0.276 (Agilent).

### Activity of mitochondrial respiratory complexes

For cell permeabilization experiments, cells were seeded in XFe96 plates, as described before in the previous section, and treated with DMSO or 1 μM of drug treatment for 24 h. Cell medium was removed, and manitol and sucrose buffer (MAS-BSA) was added (MAS: 220 mM mannitol, 70 mM sucrose, 10 mM KH_2_PO_4_, 5 mM MgCl_2_, 2 mM HEPES, 1 mM EGTA, 4 mg/mL fatty acid free BSA, pH = 7.20). Cells in MAS buffer were incubated at 37 °C in the absence of CO_2_ for 10 min. Baseline OCR measurements were recorded before administration of permeabilizer (saponin, SAP: 40 μg/mL) and complex substrate (complex I: 10 mM pyruvate, 5 mM malate; complex II: 10 mM succinate, 1 μM rotenone; complex IV: 0.5 mM TMPD, 2 mM ascorbate). ADP (1 mM) was also added in all conditions and pH was adjusted to 7.2. Maximal respiration was assessed before treatment with oligomycin A (1 μM) and finally complex inhibitors were injected to abolish respiration (complex I: 1 μM rotenone, complex II: 2 μM antimycin A, complex IV: 20 mM azide). OCR measurements were normalized to protein O.D. as described before. Data analysis was conducted with the Seahorse Wave software version 2.2.0.276 (Agilent).

### Extracellular metabolite analysis

A total of 50,000 cells/well were seeded in 12-well plates. Next day, media was replaced with fresh media (800 μL), with either DMSO or treatment, and cell density was measured (*t* = 0). After 24 h of treatment, media was collected and cell number was determined in each well (*t* = 24). Media collected was kept at −80 °C until samples were run. Two hundred microlitres of each sample were transferred to 96-well plates and concentration of glutamate, glucose, lactate and glutamine was determined with a 2950 Biochemistry Analyzer (YSI). Exchange rates were calculated and normalised to cell number. Three technical replicates were used in every independent experiment. At least three independent experiments were performed.

### Metabolite analysis by LC–MS

On ice, cells were washed three times in cold PBS before adding 500 μL of extraction buffer (methanol, acetonitrile, and water (5:3:2) v/v) at 4 °C. After 5 min at 4 °C, buffer was transferred to Eppendorf tubes and spun for 10 min, 12,000 rcf at 4 °C. Supernatants were transferred into HPLC vials and stored at −80 °C prior to LC–MS analysis. Metabolite analysis was performed using LC–MS as described in ref. ^38^. Briefly, polar metabolites in the samples were measured using HILIC chromatography and full scan mass spectrometry on a Thermo Q Exactive mass spectrometer. Commercial standards of all detected metabolites were run on the system prior to analysis. Peak areas of the metabolites were identified by a combination of mass and retention time using Thermo TraceFinder software. Peak areas of each sample were normalized to amount of protein. Quantification of protein was performed by the Modified Lowry method according to the manufacturer’s protocol. Representation of normalized absolute or relative peak areas was performed using GraphPad Software Prism 6 or Metabolite AutoPlotter (https://mpietzke.shinyapps.io/AutoPlotter/). Three technical replicates were performed per experiment.

### Transmission EM

Cells were fixed in 0.1 M sodium cacodylate buffer containing 2.5% glutaraldehyde, during 10 min at RT and then 1 h at 4 °C. Post fixation was performed with 1% osmium tetroxide/0.1 M sodium cacodylate buffer for 1 h in the dark. Then, en bloc stained with 0.5% aqueous uranyl acetate for 1 h in the dark and processed in a standard manner. Samples were fresh resin embedded in flat bed moulds and left for polymerisation. Then, embedded samples were cut in 60–70 nm ultrathin sections (LEICA Ultra Cut UCT) and contrast stained with 2% methanolic uranyl acetate during 5 min and Reynolds lead citrate during 5 min. Samples were viewed on a JEOL 1200EX transmission electron microscope (TEM) at an accelerating voltage of 80 kV. TIF images were captured using a 2Kx2K digital camera (Cantega) and iTEM (Olympus) as the software imaging system. A random selection of at least 20 mitochondria across different cells and fields was analysed for each condition. Then, mitochondrial area was quantified using ImageJ software.

### Blue native polyacrylamide gel electrophoresis

Cells were pelleted and resuspended in NativePAGE Sample Buffer (1x) (Thermo Fisher Scientific, BN2003) with 1% digitonin. Then, centrifuged at 20,000 × *g* for 30 min at 4 °C. Protein concentration in supernatant was determined by BCA protein assay and then samples were kept at −80 °C. One hundred micrograms of protein lysates were loaded into NativePAGE 3–12% Bis-Tris Protein Gels (Thermo Fisher Scientific, BN1001BOX). Electrophoresis and consequent western blotting were performed using the NativePAGE Novex Bis-Tris Gel System (Thermo Fisher Scientific) as described in the manufacturer’s protocol. After transfer, membrane was incubated in 8% acetic acid for 15 min to fix the proteins. Then, blocking, washing and primary antibody incubation steps were performed as described previously on “Western blotting” section. After primary antibody incubation, membranes were incubated with ECL mouse IgG, HRP-antibody (GE Healthcare Life Sciences, NA931). Proteins bands were detected using Clarity Max Western ECL Substrate (Bio-Rad, 1705062) and ChemiDoc Imaging System (Bio-Rad, 17001401).

### Statistical analysis

All data are represented as mean ± SEM. Samples were compared using two-tailed, unpaired Student’s *t* test, *p < 0.05, **p < 0.01, ***p < 0.001 and ****p < 0.0001. Statistical analysis was performed using Prism version 6.0c (GraphPad Software, La Jolla, CA).

## Supplementary information

Supplemental Figure Legends

Supplemental Figure 1

Supplemental Figure 2

Supplemental Figure 3

Supplemental Figure 4

Supplemental Figure 5
