# Supplementary material for: Venetoclax causes metabolic reprogramming independent of BCL-2 inhibition
Source: Cell Death Dis. 2020 Aug 13;11(8):616. doi: 10.1038/s41419-020-02867-2 (PMC7426836; doi:10.1038/s41419-020-02867-2)
Supplement: Supplementary file 1 — Supplemental Figure Legends [file 41419_2020_2867_MOESM1_ESM.docx]

**Supplemental Figure Legends**

**Figure S1. Venetoclax affects metabolism independent of BAX, BAK mediated cell death**

**A**. CT26 cells were treated with venetoclax (1 μM) for 24 hours and then OCR was measured by Seahorse XF-96 Analyzer. Data represent the mean of three independent experiments ± SEM. **B**. OCR of CT26 cells was monitored over 4 hours after addition of venetoclax (1 μM). Data represent the mean of ten technical replicates from one representative experiment. **C.** CT26 CRISPR-EMPTY or CRISPR-BAX/BAK cells were treated with ABT-737 (10 μM) and S63845 (1 μM) alone or in combination for 72 hours. Cell viability was monitored with Sytox Green dye exclusion and IncuCyte live cell imaging. Data represent the mean of two technical replicates. (*) shows decrease of OCR. OA: oligomycin A, AA: antimycin A, Rot: rotenone.

**Figure S2. Venetoclax inhibits the TCA cycle causing reductive carboxylation**

**A**. CT26 cells were treated for 24 hours with venetoclax (1 μM) and cultured with labelled glutamine (^13^C-GLN). Intracellular metabolites were extracted and analysed by LC-MS. Relative peak area of one independent experiment (out of two) with three technical replicates +/- SEM. **B**. CT26 cells were treated for 24 hours with venetoclax (1 μM) and cultured with labelled glucose (^13^C-GLUC). Intracellular metabolites were extracted and analysed by LC-MS. Graphs represent relative peak area of three technical replicates +/- SEM. Graphs were generated using Metabolite AutoPlotter.

**Figure S3. The metabolic effects of venetoclax are independent of BCL2 family proteins**

**A**. CT26 cells were treated for 24 hours with S55746 (1 μM) and cultured with labelled glutamine (^13^C-GLN). Intracellular metabolites were extracted and analysed by LC-MS. Graphs represent total and relative peak area of three technical replicates +/- SEM. Graphs were generated using Metabolite AutoPlotter. **B**. BCL-2 protein expression in SVEC CRISPR-EMPTY and SVEC CRISPR-BCL2 cells was determined by western blot, α-tubulin was probed as a loading control. OCR measured by Seahorse XF-96 Analyzer (data represent the mean of one experiment with five technical replicates) and metabolites levels measured by LC-MS in SVEC CRISPR-EMPTY and SVEC CRISPR-BCL2 cells (data are the mean of three technical replicates). **C**. BCL-2 protein expression in CT26 cells following transfection with non-targeting siRNA (NTC) or siRNA against BCL-2 was determined by western blot, α-tubulin was probed as a loading control. NTC or BCL-2 siRNA transfected CT26 cells were treated with venetoclax (1 μM) for 24 hours and assessed for basal OCR; data are the mean of three independent experiments +/- SEM (**D**) and succinate levels; data are the mean of one representative experiment (out of two) with three technical replicates (**E**). **F**. BCL-2 protein expression in B16F10 CRISPR-EMPTY and B16F10 CRISPR-BCL2 cells was determined by western blot, α-tubulin was probed as a loading control. B16F10 cells were treated with venetoclax (1 μM) for 24 hours and assessed for basal OCR; data are the mean of three independent experiments +/- SEM (**G**) and intracellular metabolite levels; data are the mean of one independent experiment with three technical replicates (**H**). Samples were compared using two-tailed, un-paired Student’s t-test *p<0.05, **p<0.01, ***p<0.001.

**Figure S4. Venetoclax affects mitochondrial morphology and function**

TEM images from MCF7 cells after 24 hours of venetoclax (1 μM) treatment. Scale bar = 1 μM. A random selection of at least 20 mitochondria across different cells and fields was analysed for each condition from images in A. Mitochondrial area (in μm^2^) was quantified using ImageJ software. Data are the mean of at least 20 mitochondria +/- SEM.

**Figure S5**. **Venetoclax affects metabolism dependent on ATF4.**

MCF7 cells were treated with either control treatment or venetoclax (1 μM) for 24 hours. Complex I, II, III, IV and V expression was analysed by BN-PAGE. HSP60 was included as a loading control. One representative experiment out of three independent experiments. Ven = venetoclax.
