# Supplementary figures and images for: Venetoclax causes metabolic reprogramming independent of BCL-2 inhibition
Source: Cell Death Dis. 2020 Aug 13;11(8):616. doi: 10.1038/s41419-020-02867-2 (PMC7426836; doi:10.1038/s41419-020-02867-2)

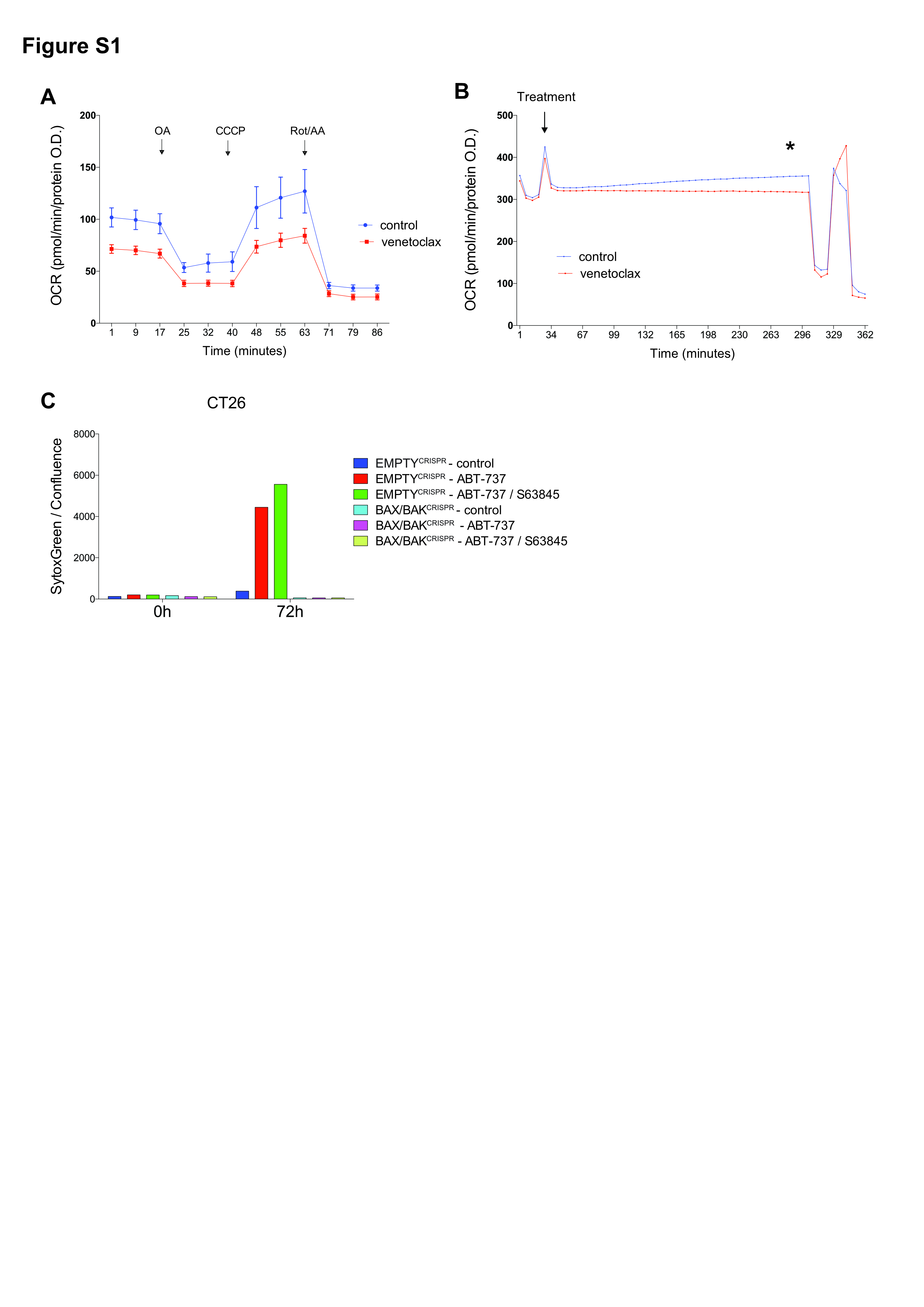

Supplement: Supplementary file 2 — Supplemental Figure 1 [file 41419_2020_2867_MOESM2_ESM.tif]

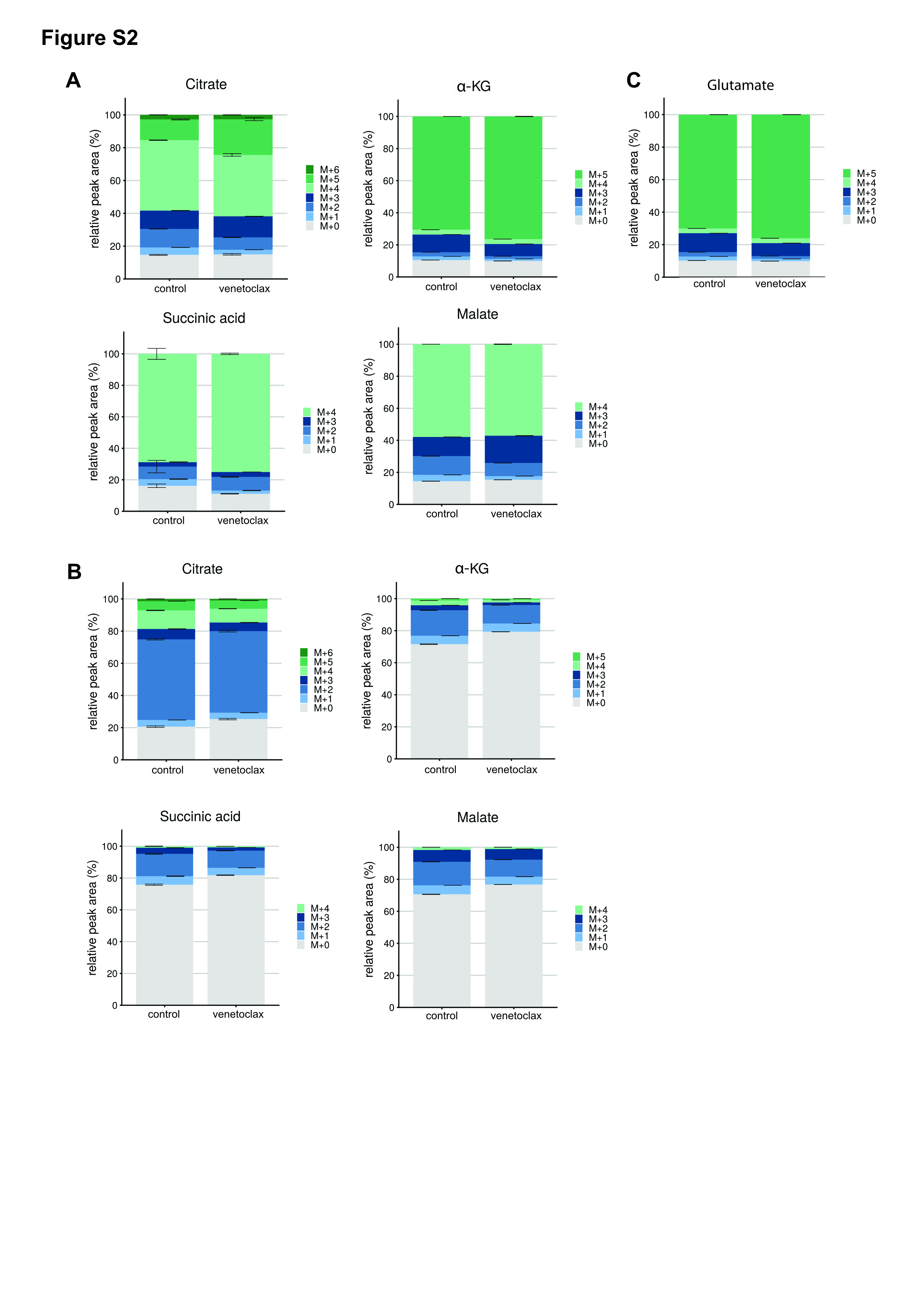

Supplement: Supplementary file 3 — Supplemental Figure 2 [file 41419_2020_2867_MOESM3_ESM.tif]

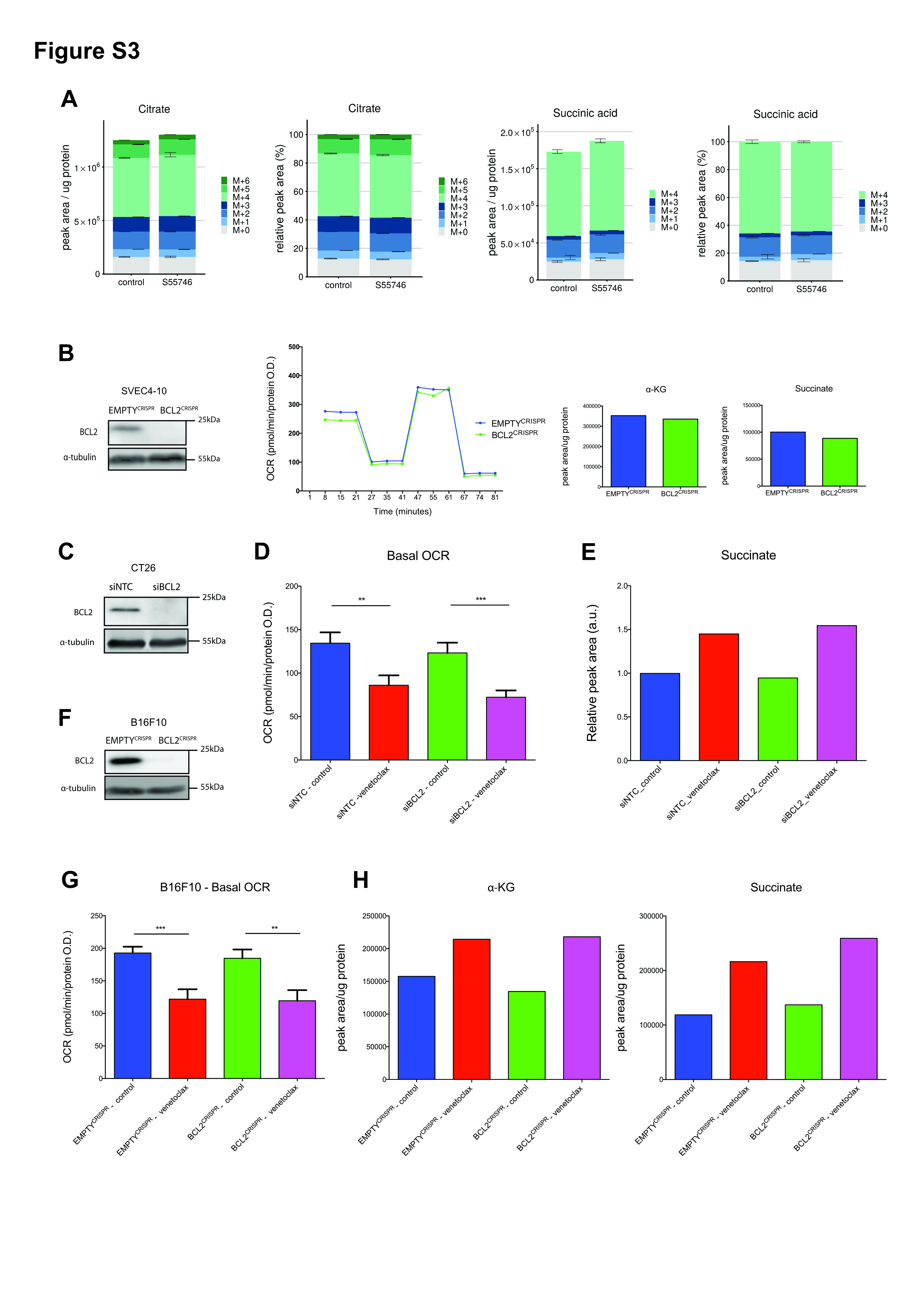

Supplement: Supplementary file 4 — Supplemental Figure 3 [file 41419_2020_2867_MOESM4_ESM.tif]

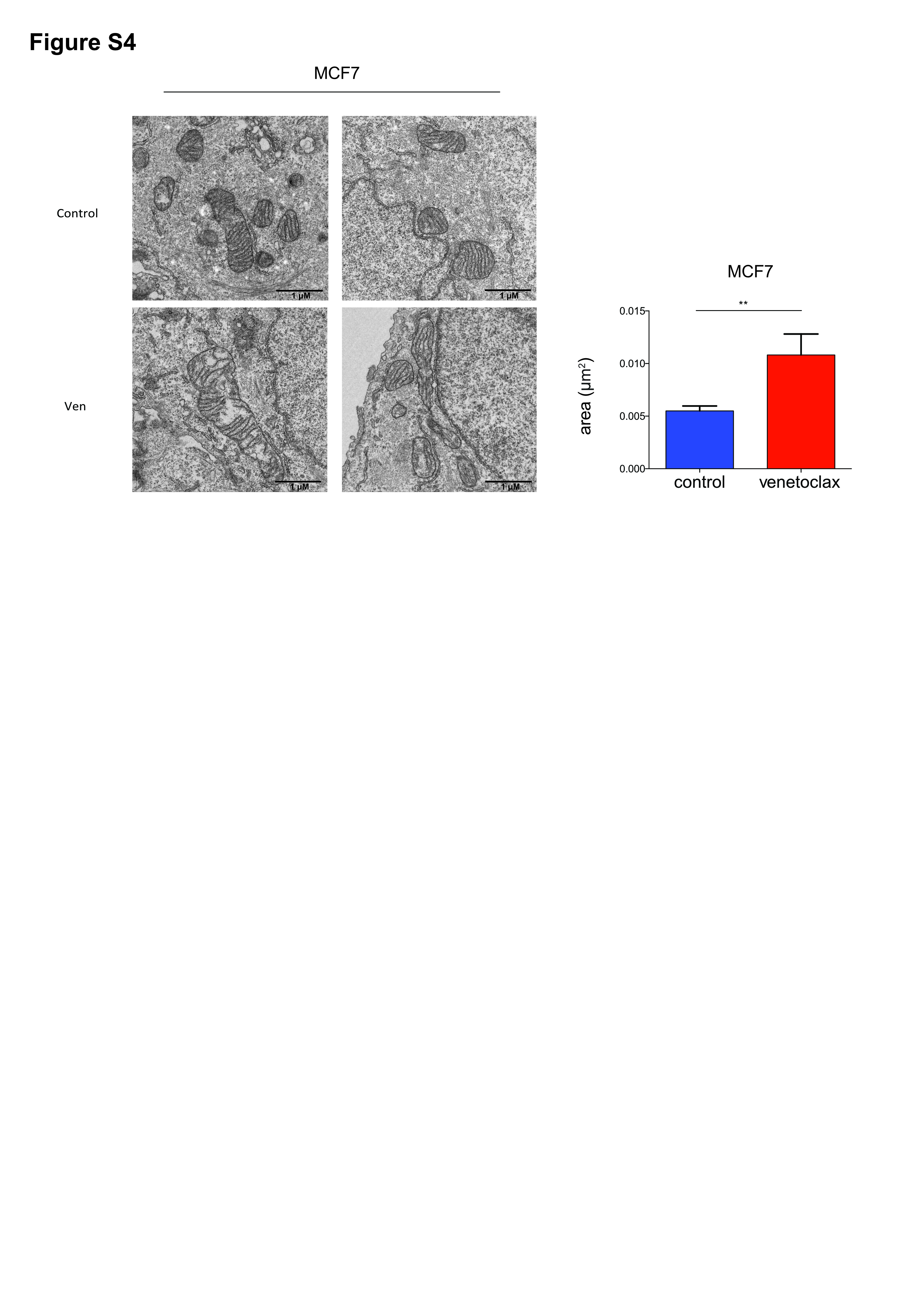

Supplement: Supplementary file 5 — Supplemental Figure 4 [file 41419_2020_2867_MOESM5_ESM.tif]

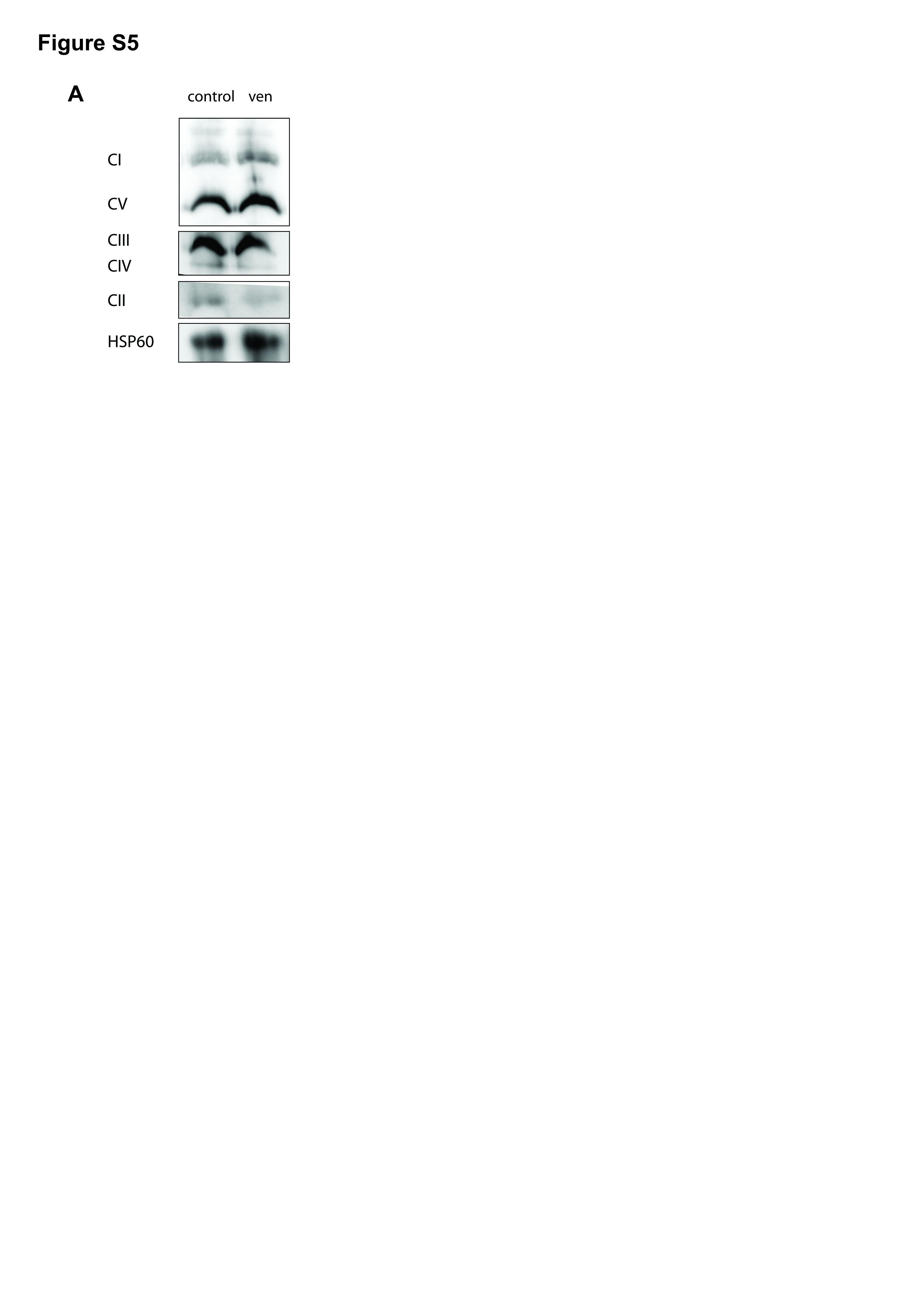

Supplement: Supplementary file 6 — Supplemental Figure 5 [file 41419_2020_2867_MOESM6_ESM.tif]
